# 5G remote-controlled snake-arm single-port robotic pneumovesical Cohen ureteroneocystostomy: first animal study

**DOI:** 10.3389/fped.2026.1760104

**Published:** 2026-05-19

**Authors:** Shengqi Zheng, Shouyue Chen, Xu Cui, Chaoming Zhou

**Affiliations:** 1 College of Clinical Medicine for Obstetrics & Gynecology and Pediatrics, Fujian Medical University, Fuzhou, Fujian Province, China; 2 Pediatric Urology, Fujian Children’s Hospital (Fujian Branch of Shanghai Children’s Medical Center), Fuzhou, Fujian Province, China; 3 Outpatient Department, Chinese People’s Armed Police Force Fujian Provincial Corps Hospital, Fuzhou, Fujian Province, China

**Keywords:** 5G telesurgery, Cohen procedure, pneumovesical technique, robotic-assisted surgery, ureteral reimplantation

## Abstract

**Background:**

To explore the initial technical feasibility of 5G remote-controlled single-port robotic-assisted pneumovesical ureteroneocystostomy (Cohen procedure) in an animal model, and to provide preliminary experimental evidence for this integrated surgical approach.

**Methods:**

A healthy female juvenile Landrace crossbred pig (3–4 months old, 32 kg) was used as the experimental model. The procedure was performed at the Animal Experimental Center of Surgerii Robotics Co., Ltd., with remote control via 5G network from Fujian Children's Hospital. A snake-arm single-port robotic system was utilized to perform pneumovesical ureteroneocystostomy (Cohen procedure). Technical feasibility and parameters were evaluated.

**Results:**

The surgery was successfully completed with total operative time of 92 min and blood loss of 6 mL. The 5G network maintained stable video transmission and real-time control throughout the procedure. No major intraoperative complications occurred, and the animal recovered uneventfully during the 24-hour observation period. Anatomical examination confirmed accurate ureteral reimplantation, patent anastomosis, and absence of urinary extravasation.

**Conclusions:**

This study successfully implemented 5G remote-controlled snake-arm single-port robotic-assisted pneumovesical ureteroneocystostomy (Cohen procedure) in an experimental animal model for the first time. The single-case piglet model demonstrated the initial technical feasibility and provided a proof-of-concept for its procedural feasibility under experimental conditions. It provides important technical support for minimally invasive treatment of vesicoureteral reflux in children, with promising potential for future research.

## 1 Introduction

Vesicoureteral reflux (VUR) is one of the most common congenital malformations of the urinary system in children. The prevalence rate is approximately 1%–3% in the general pediatric population, while it can be as high as 30%–50% in children with a history of recurrent febrile urinary tract infections (1). The disease is characterized by the abnormal reflux of urine from the bladder to the ureters and even the kidneys. Without timely intervention, it may lead to recurrent urinary tract infections, renal scarring, and in severe cases, progress to hypertension, renal insufficiency, or even end-stage renal disease (2). Although traditional open surgery has a definite therapeutic effect, it has disadvantages such as large surgical trauma and slow postoperative recovery (3). With the development of minimally invasive technology, pneumovesical laparoscopic ureteroneocystostomy has become one of the main surgical methods for the treatment of moderate to severe VUR, which is performed by insufflating CO_2_ gas into the bladder to establish an operative space (4). Despite the advantages of minimal trauma and rapid recovery, the traditional laparoscopic pneumovesical Cohen technique remains technically challenging, primarily due to the need for delicate suturing in a narrow space, limited operative space from the small bladder capacity, relatively long surgical time, and the “chopstick effect” that further increases operational difficulty (5). The development of robot-assisted surgery provides a new possibility for solving these technical problems, with advantages such as high operational flexibility, strong stability, and clear visual field (6). Studies have shown that robot-assisted ureteral reimplantation is superior to traditional open surgery in terms of surgical success rate and postoperative complication control (7).

Although robotic technology is relatively mature for extravesical ureteral reimplantation in children, intravesical procedures remain challenging due to limited operating space. This disparity has driven innovations in robotic design, notably single-port systems. Single-port robots address the critical issue of space constraints through their compact design and enhanced maneuverability, while also offering additional benefits including minimized surgical trauma, improved cosmetic outcomes, and reduced postoperative pain (8, 9). Furthermore, the integration of fifth-generation (5G) communication technology represents a significant extension of robotic surgical capabilities. With its ultra-low latency, high bandwidth, and ubiquitous connectivity features, 5G technology provides a robust infrastructure for remote surgical applications (10). Combined with advanced robotics, this approach resolves physical limitations of pediatric intravesical surgery and overcomes geographical barriers through state-of-the-art communication.

While robotic surgery is widely used in pediatric urology, integrating 5G remote control with snake-arm single-port robotic pneumovesical surgery remains exploratory. Animal experiments are essential for developing new clinical technologies. Pigs have a urinary system anatomy highly similar to humans, making them ideal models for studying urological diseases and surgical techniques (11, 12). Juvenile pigs, with body size and organ development comparable to children, accurately simulate pediatric surgical conditions. This study used a juvenile pig model to explore the initial procedural execution and technical feasibility as a proof-of-concept for 5G remote-controlled single-port robotic-assisted pneumovesical ureteroneocystostomy (Cohen procedure).

## 2 Materials and methods

### 2.1 Experimental animal selection

One healthy female juvenile Landrace crossbred pig (identification number: SP0426), aged 3–4 months and weighing 32 kg, was selected for this study. The experimental animal was provided by Jiangxi Yinshe Biotechnology Co., Ltd., with an experimental animal quality certificate numbered NO20251020001. Experimental operations were conducted by professionally trained physicians and technical personnel to ensure adequate protection of animal welfare.

### 2.2 Configuration of 5G remote surgery system

This study utilized the SHURUI snake-arm single-port robotic system (Surgerii Robotics Co., Ltd., Beijing, China), an NMPA-approved platform for endoscopic urological procedures. The system is mainly composed of a surgeon console, surgical operation platform, image processing system, snake-arm, and 3D high-definition flexible electronic endoscope. The surgeon console, serving as the main operating interface, is equipped with a 27-inch 3D display, force feedback operation handles, and multi-functional foot switches. The surgical operation platform includes three robotic arm systems, a surgical instrument library, and an image processing unit. The robotic snake-arm is designed with excellent flexibility, and its end effector can achieve multi-directional movement, enabling precise surgical operations in confined spaces.

Remote surgical connection was established using 5G technology combined with a dedicated wired internet line over public network infrastructure, achieving a highly stable average end-to-end surgical latency of 28 ms and a packet loss rate below 0.001%. The remote control platform was based on the H.265/HEVC video coding standard, supporting 4 K (3840 × 2160 pixels) resolution and 30fps frame rate, with a dynamic video bitrate adjustment range of 2–20Mbps. To ensure data transmission security, the system used Advanced Encryption Standard (AES-256) for data encryption and established an emergency mechanism with automatic fault detection and backup link switching. The Fujian end established secure connections with the surgical platform in the animal room through a dedicated China Telecom 5G medical private network utilizing 5G network slicing technology and 5G customer premises equipment. The real-time network performance monitoring system updated latency data every 100 ms to ensure signal stability. Importantly, these parameters reflect an ideal laboratory setup utilizing a dedicated 5G medical private network, which provides a critical technical baseline but may differ from variable clinical environments.

### 2.3 Surgical method

Robotic equipment at both the remote and animal room ends was activated 1 h preoperatively for system self-test and network connection stability testing. Network latency and packet loss rate of control signals were real-time monitored via User Datagram Protocol to ensure network connectivity met the requirements for remote surgery. Instrument function tests included robotic arm movement range verification, force feedback calibration, and high-definition image transmission quality assessment.

**Figure 1:**
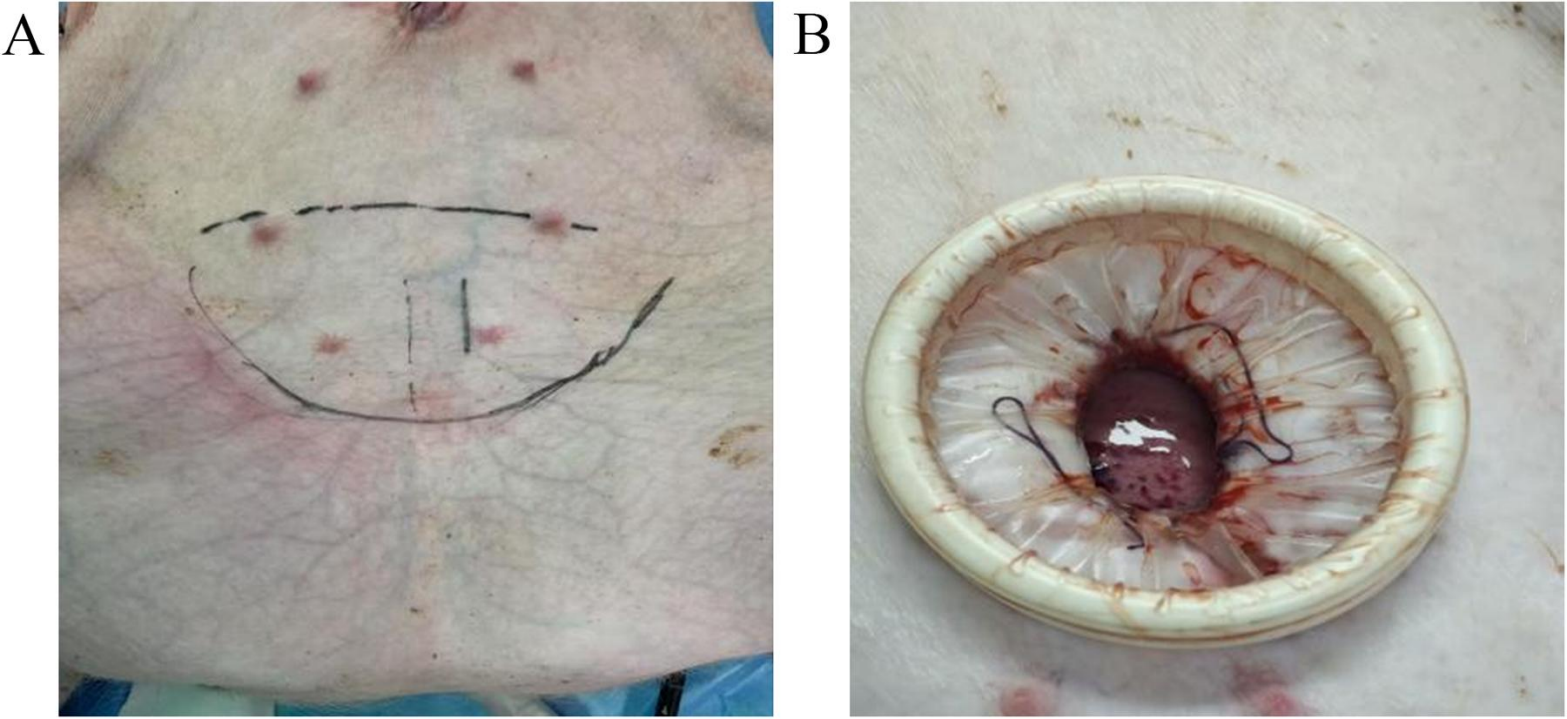
Incision design and pneumovesical channel establishment **A**: bladder and abdominal incision positioning; **B**: single-port channel establishment.

After fasting, animals were anesthetized, intubated, monitored, and positioned for surgery. The bladder puncture site was identified using anatomical landmarks (Figure 1A). A puncture needle was inserted vertically, its angle adjusted, and then advanced toward the pelvic cavity. Successful bladder entry was confirmed by aspirating urine, following which the needle was connected to a CO_2_ insufflator. CO_2_ was insufflated at a flow rate of 2 L/min with an initial pressure setting of 8 mmHg, and a total insufflation volume of approximately 150–200 mL to fully distend the bladder, resulting in obvious abdominal distension. A longitudinal incision of approximately 3 cm was made in the abdominal wall. The skin, subcutaneous fat tissue, and anterior rectus sheath were incised, followed by incision of the anterior bladder wall. The anterior bladder wall was fixed to the abdominal wall to ensure stable positioning of the bladder during surgery. A 25 mm single-port access kit was inserted through the incision to establish the single-port operating channel (Figure 1B). A pressure monitoring sensor was connected to maintain the intravesical pressure within the range of 8–10 mmHg.

A 3D high-definition flexible endoscope was inserted and connected to the image processing system to confirm clear visualization inside the bladder and intact bladder wall without injury. The remote surgeon controlled the robotic system via 5G wireless network and precisely adjusted the robotic arm position according to the preoperative surgical plan. Snake-arm surgical instruments were inserted through the single-port channel, and master-slave calibration was performed to ensure complete consistency between the robotic arm movement trajectory and the operating handle movements.

**Figure 2:**
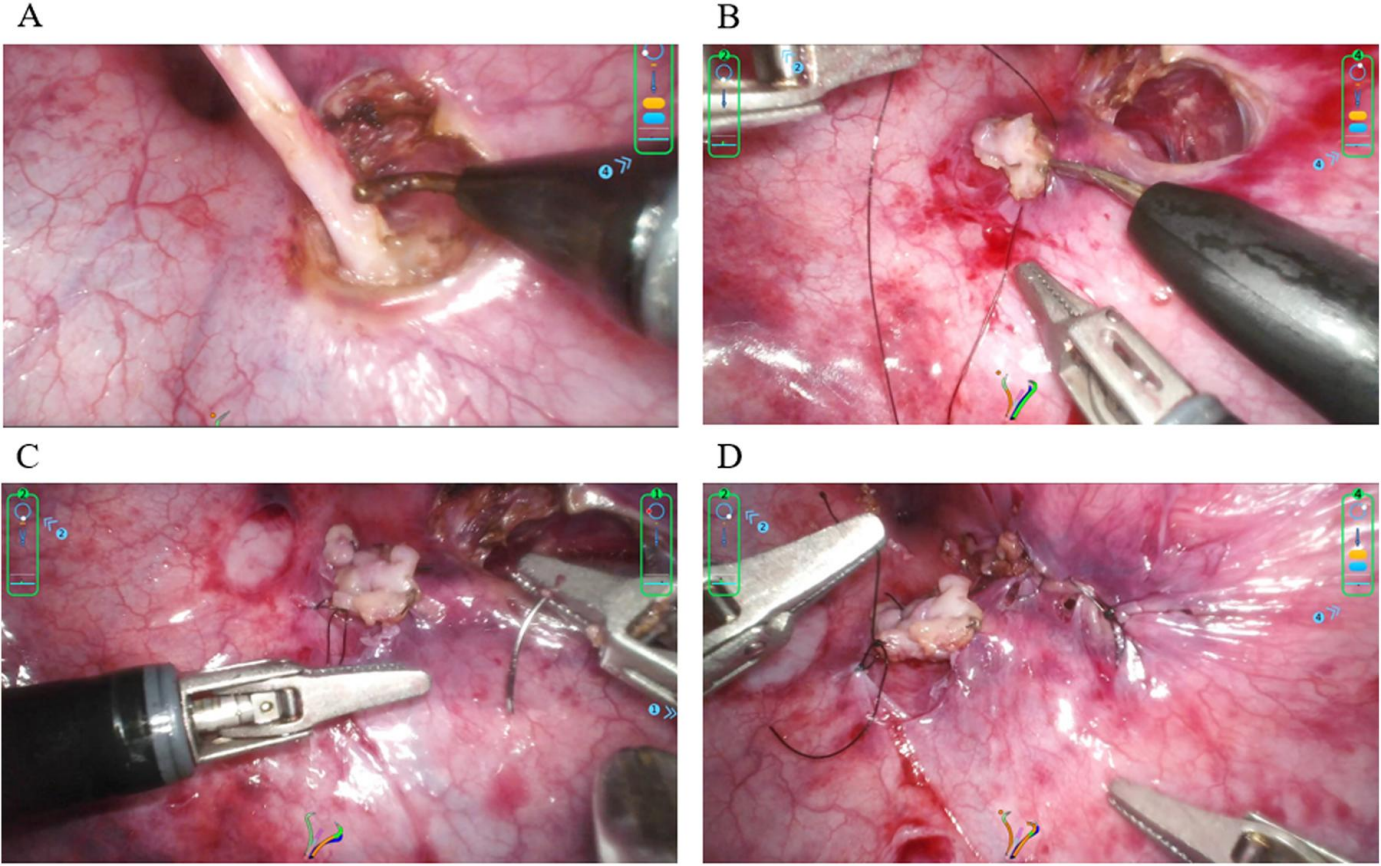
Ureteral dissection and reimplantation **A**: circumferential ureteral dissection; **B**: submucosal tunnel creation and ureteral transposition; **C**: mucosal and ureteral orifice suturing; **D**: bladder wall closure and hemostasis.

The Cohen procedure started with precise localization of the ureteral orifices under endoscopic visualization. A circumferential mucosal incision was made using an electrocautery hook to fully expose the ureteral end. The ureter was mobilized 4–6 cm along its course, with careful preservation of ureteral blood supply (mainly from vesical and internal iliac artery branches) (Figure 2A). The ureteral end was appropriately trimmed according to the degree of dilation to restore normal diameter. A 1 cm mucosal incision was made approximately 1.5 cm above the contralateral ureteral orifice, and submucosal tissue was carefully dissected using a separation hook to create an adequate submucosal tunnel. The tunnel length was approximately 1.5 cm with a width twice the ureteral diameter. A 5:1 tunnel length-to-diameter ratio was maintained to effectively prevent postoperative reflux. The ureter was pulled through the submucosal tunnel to the new orifice position and secured to the bladder mucosa using interrupted sutures (Figures 2B,C). Tension-free positioning of the ureter within the tunnel was ensured with watertight anastomosis, and the original ureteral hiatus was closed with continuous sutures (Figure 2D).

Intraoperative quality checks included bladder saline instillation to test anastomotic integrity, observation of ureteral peristalsis and urine drainage, and careful inspection of the surgical field for active bleeding points. At the conclusion of the procedure, CO_2_ gas was slowly released from the bladder to avoid physiological discomfort caused by sudden pressure drop. Abdominal incisions were closed in anatomical layers, and pelvic drainage tubes and urinary catheters were placed.

## 3 Results

### 3.1 Surgical outcome and 5G network performance

The surgery was successfully completed without conversion to open surgery. The total operative time was 92 min, with minimal intraoperative blood loss of 6 mL, primarily from capillary oozing during cystotomy and ureteral dissection, which was effectively controlled using electrocoagulation. No blood transfusion or special hemostatic measures were required throughout the procedure.

The 5G network demonstrated excellent performance during the surgery, with video transmission quality reaching 4 K resolution at a stable frame rate of 30 fps without any lag. Control signal response was excellent, with no significant delay observed between the surgeon's hand movements at the remote console and the robotic arm movements. The 5G network connection remained stable throughout the entire surgical procedure, without network interruption or significant latency increase.

### 3.2 Technical feasibility assessment

The snake-arm single-port robotic system exhibited outstanding intravesical manipulation capabilities in juvenile pigs, with its 7-DOF design providing exceptional dexterity in the limited bladder space. The repeat positioning accuracy of the robotic arms was measured at ± 0.08 mm, significantly exceeding the precision requirements for clinical surgical procedures. Intravesical pressure was successfully maintained within the range of 8–10 mmHg, with no gas leakage or abnormal pressure fluctuations observed during the operation.

The single-port design successfully enabled simultaneous operation of three interchangeable 8.4 mm snake-arm instruments and one endoscope through a small incision. No mechanical arm collisions occurred between instruments, and no surrounding mucosal tissue tearing or bleeding was observed due to instrument manipulation. The flexible movement characteristics of the snake-arm instruments effectively compensated for the inherent limitations of the single-port design in terms of operating angles, ensuring smooth progression of the surgical procedures. The average instrument change time was 30 s completed through the quick-change channel of the single-port trocar without compromising the continuity of the surgical workflow.

The main surgical technical challenges included difficulty in ureteral dissection, challenges in constructing the submucosal tunnel, and technical difficulties in anastomotic suturing. This study employed the snake-arm single-port robotic system, utilizing interchangeable 8.4 mm instruments, which effectively resolved these challenges through its multi-degree-of-freedom movement capabilities. For dissecting the slender ureter (2–3 mm) of juvenile pigs, the 8.4 mm micro fine forceps provided adjustable clamping force of 0.1–5N, enabling gentle grasping without tissue damage. The right snake-arm dissector adopted a blunt dissection strategy with a 7-degree-of-freedom design suitable for operation in confined spaces, and when combined with 20W low-power electrocoagulation, reduced the risk of thermal injury. In constructing the bladder submucosal tunnel, the blunt characteristics of the snake-arm dissector effectively protected the fragile mucosa, while the distal pressure sensor continuously monitored tissue contact force. The multi-degree-of-freedom movement enabled three-dimensional precise control, accurately adjusting the tunnel width to twice the diameter of the ureter, ensuring sufficient space while avoiding excessive dissection. For addressing the suturing challenges in confined spaces, the 7-degree-of-freedom snake-arm enabled multi-angle operation, and precise motion control minimized the number of sutures. Combined with intracorporeal knot-tying technology and real-time force feedback, optimal tension at the anastomosis was maintained.

### 3.3 Immediate postoperative observation (24 h)

Immediate postoperative anatomical examination revealed accurate ureteroneocystostomy positioning, patent anastomosis, and no urine extravasation. The bladder wall maintained good integrity with no significant damage. Ureteral blood supply was adequate with no signs of ischemia. The animal showed excellent postoperative anesthetic recovery, with a time of 28 min from drug discontinuation to full awakening. The recovery process was smooth without anesthesia-related complications such as agitation or vomiting. The animal started consuming liquid diet at 6 h postoperatively and resumed normal diet at 12 h postoperatively with good appetite. Normal activity levels, including standing, walking, and drinking, were restored within 24 h postoperatively.

## 4 Discussion

This study successfully performed remote-controlled snake-arm single-port robotic-assisted pneumovesical ureteroneocystostomy in a juvenile pig model for the first time. By combining 5G remote control technology, snake-arm single-port robotics, and pneumovesical surgery, this approach creates a synergistic method that enhances surgical precision, limits surgical trauma, and enables remote operation capabilities. The ultra-low latency of 5G technology, which is far below human visual reaction time, provided reliable support for real-time remote surgical control and stable procedural execution. This technological combination offers a promising solution for complex urological procedures in children.

Juvenile pigs were used as experimental models in this study to simulate the anatomical and physiological characteristics of children. Juvenile pigs share similarities with children in multiple aspects, including anatomical structure, physiological function, and tissue properties. The kidney weight, volume, and ureteral length of juvenile pigs are comparable to those of human children, making the surgical difficulty and technical requirements similar to pediatric surgery (13). The tissue elasticity, vascular distribution, and healing capacity of juvenile pigs are similar to those of children, facilitating the evaluation of surgical technique applicability and technical feasibility (14, 15).

As a minimally invasive surgical approach, pneumovesical technology demonstrated significant procedural utility in this study. Insufflation of the bladder cavity creates a stable operative space, facilitating bladder wall distension and tissue stratification, improving visualization, and reducing traction and mechanical injury to the detrusor muscle, thereby lowering the risk of postoperative voiding dysfunction (16, 17). Compared with the traditional transabdominal approach, pneumovesical technology significantly reduces abdominal organ manipulation and associated adhesions (18).

Bladder injury is a significant complication of pneumovesical surgery (19). In this study, although clinical pediatric pneumovesical procedures typically use percutaneous suspension, a longitudinal incision was made in the anterior bladder wall under direct visualization, and the anterior bladder wall was fixed to the abdominal wall to overcome the disadvantage of much higher bladder mobility in young pigs compared to humans, ensuring stable positioning of the bladder during surgery and effectively establishing a single-port operating channel. However, the necessity of an open cystotomy and bladder fixation in this model differs from standard pneumovesical minimally invasive techniques used in humans, indicating that future procedural refinements will be required for clinical translation.

The main limitations of previous pediatric pneumovesical ureteroneocystostomy include insufficient instrument flexibility due to limited operating space within the bladder cavity, restricted field of view with traditional endoscopes, instrument designs not fully adapted to the requirements of delicate intravesical operations, and special technical challenges posed by the smaller anatomical structures of pediatric patients (17). The snake-arm single-port robotic system effectively addresses these limitations with its unique design features. This system utilizes a multi-degree-of-freedom snake-arm robotic design, which provides highly flexible operational capabilities within the limited bladder cavity, effectively addressing the problem of restricted operating angles with traditional laparoscopy in confined spaces (20). The fine articular mobility and tremor filtering capabilities of the snake-arm enable excellent performance in delicate procedures such as ureteral dissection and mucosa-to-mucosa anastomosis, significantly improving surgical precision (21). Clinical studies have shown that the snake-arm single-port robotic system achieves favorable outcomes in complex surgeries including pediatric pyeloplasty and adult radical prostatectomy, with high surgical success rates and low complication rates (22, 23). Its 7 cm minimum working distance (compared to 10 cm for the da Vinci SP) facilitates adequate instrument triangulation within the severely confined pediatric pneumovesicum (8).

Under these experimental conditions, the robotic system demonstrated notable technical precision. The three-dimensional high-definition visualization and the snake-arm design provided excellent maneuverability with tremor filtering in confined spaces (24). Furthermore, the single-port approach facilitated a small incision, resulting in an intraoperative blood loss of only 6 mL.

This technology could potentially be explored for the treatment of vesicoureteral reflux as well as other pediatric urological conditions such as megaureter, and ureterocele (25). From a clinical perspective, single-incision approaches aim to achieve potential benefits in pediatric patients. By using the natural umbilical folds as the surgical entry site, this technique can achieve an almost scarless cosmetic outcome, minimize damage to the skin and fascia, and significantly improve cosmetic appearance and parental satisfaction. Furthermore, 5G remote surgery technology has significant implications for optimizing healthcare resource allocation. The uneven distribution of medical resources is a prevalent challenge worldwide, with high-quality medical resources often concentrated in medical centers while underdeveloped regions have relatively limited access (26). This technological approach enables medical experts to provide direct surgical services to patients at distant locations, reducing the time and economic burden of patient referrals and improving healthcare accessibility (27).

This study has several limitations. The single-case design (*n* = 1) without a control group precludes statistical analysis, the determination of true complication rates, and the assessment of reproducibility, serving strictly as an initial proof-of-concept. Furthermore, the observation period was limited to the acute 24-hour postoperative phase, making it impossible to evaluate long-term outcomes such as ureteral patency, reflux recurrence rate, and renal function changes. Although juvenile pigs share anatomical and physiological characteristics with children, there are still some differences. Additionally, this technology integrates multiple complex technologies including 5G remote control, robotic surgery, and pneumovesical technology, requiring high-end equipment and skilled personnel, which may pose challenges for widespread adoption.

Future research should reduce network latency, integrate AI to improve precision, expand animal studies, conduct multicenter trials, combine with VR for training, and establish standards, training systems, and ethical frameworks.

## 5 Conclusion

This single-case study demonstrated the initial technical operability and served as a proof-of-concept for 5G remote-controlled snake-arm single-port robotic pneumovesical ureteroneocystostomy in juvenile pigs as pediatric models. Under experimental conditions, the technology demonstrated technical precision and remote accessibility. 5G low latency and robotic stability ensure reliable remote surgery. Findings provide evidence for pediatric urology minimally invasive surgery, demonstrating potential for tele-surgical applications. Future research should conduct large-sample clinical studies for standardization and translation.

## Data Availability

The original contributions presented in the study are included in the article/Supplementary Material, further inquiries can be directed to the corresponding author.
